# Comparison between Dual-Energy X-ray Absorptiometry and Bioelectrical Impedance Analyses for Accuracy in Measuring Whole Body Muscle Mass and Appendicular Skeletal Muscle Mass

**DOI:** 10.3390/nu10060738

**Published:** 2018-06-07

**Authors:** Seo Young Lee, Soyeon Ahn, Young Ji Kim, Myoung Jin Ji, Kyoung Min Kim, Sung Hee Choi, Hak Chul Jang, Soo Lim

**Affiliations:** 1 Department of Internal Medicine, Seoul National University College of Medicine and Seoul National University Bundang Hospital, Seongnam 13620, Korea; danaehyuk@naver.com (S.Y.L.); vernade@hanmail.net (Y.J.K.); hahajmj@hanmail.net (M.J.J.); kyoungmin02@gmail.com (K.M.K.); shchoimd@gmail.com (S.H.C.); janghak@snu.ac.kr (H.C.J.); 2 Department of Internal Medicine, Mediplex Sejong Hospital, Incheon 21080, Korea; 3 Division of Statistics, Medical Research Collaborating Center, Seoul National University Bundang Hospital, Seongnam 13620, Korea; ahnsoyeon@gmail.com

**Keywords:** bioelectrical impedance analysis, dual-energy X-ray absorptiometry, muscle mass

## Abstract

We evaluate the accuracy of whole body muscle mass (WBMM) and appendicular skeletal muscle mass (ASMM) assessed by bioelectrical impedance analysis (BIA) using an InBody770 machine (InBody, Seoul, Korea) referenced to dual-energy X-ray absorptiometry (DXA) in 507 people (mean age 63.7 ± 10.8 years, body mass index (BMI) 25.2 ± 3.5 kg/m^2^). Mean WBMMs measured by BIA and DXA were 49.3 ± 6.6 kg and 46.8 ± 6.5 kg in men and 36.1 ± 4.7 kg and 34.0 ± 4.8 kg in women, respectively. The respective effect sizes and 95% confidence intervals (CIs) for the difference were 2.49 (2.22–2.76) for men, and 2.12 (1.91–2.33) for women. Mean ASMMs measured by BIA and DXA were 22.1 ± 3.3 kg and 19.9 ± 3.2 kg in men, and 15.3 ± 2.5 kg and 13.5 ± 2.2 kg in women, respectively. The respective effect sizes and 95% CIs for the difference were 2.26 (2.10–2.41) for men and 1.75 (1.65–1.87) for women. The BIA clearly overestimated WBMM by 2.28 kg and ASMM by 1.97 kg compared with DXA. Using BMI, gender, and fat percentage, we derive equations that improved the residuals to <2 kg between methods from 38.29% to 85.91% for WBMM and 52.78% to 97.02% for ASMM.

## 1. Introduction

Most developed and developing countries have been facing rapid increases in the elderly population. A decrease in muscle mass or sarcopenia is an important contributor to declining health in this group [1,2,3,4]. In this situation, it is important to measure muscle mass accurately for the management of sarcopenia, which causes many metabolic disorders, physiological problems, and functional impairments that eventually lead to disability in elderly populations [5,6,7,8]. Therefore, preservation or buildup of muscle mass is becoming an important health issue for this age group, and accurate measurement of muscle mass is the first step for adequate prevention and treatment of sarcopenia.

The common imaging modalities to measure amount of muscle mass are computed tomography (CT) and magnetic resonance imaging (MRI). Both methods are useful to measure muscle cross-sectional area precisely [9,10]. However, CT is expensive and carries the risk of radiation exposure particularly for whole-body assessment. Although there is such no risk of radiation exposure in MRI, its high cost, long examination time, noise, regional variations in accessibility, and limited use with metal implants prevent its use as a routine screening method for elderly persons. By contrast, dual-energy X-ray absorptiometry (DXA) has several advantages, such as its easy applicability, low cost, and low radiation exposure [11]. Therefore, DXA is regarded as the gold standard method for analyzing body composition at the molecular level. In this method, the body is divided into three components: fat mass, lean mass, and bone mineral content [12,13,14]. DXA has been used for measuring muscle mass in several studies [15,16,17]. Body composition in a specific compartment can also be measured with recent upgraded software for DXA. However, there is still a small amount of radiation exposure. In addition, extremely obese people are not suitable for this method because they exceed weight limitations. 

Several other methods for body composition assessment, such as bioelectrical impedance analysis (BIA) and sonography, have now been developed. Among them, BIA has drawn attention because it is easily applicable, imposes no burden on subjects, involves easy to move and relatively cheap equipment, and most importantly, poses no radiation hazard [18,19]. Using BIA, information on body composition can be obtained simply with the subject in a standing position in a short time (less than 3 min). Thus, BIA is widely used not only in specialized facilities, but also in fitness and health checkup centers [20]. 

So far, several studies have been conducted to investigate the usefulness of BIA in measuring body composition, but mainly in people of European origin [16,17]. Moreover, another study with Mexican subjects had a small number of participants and used old machines that lacked accuracy [21]. However, there are not many studies using BIA with Asians. Body composition differs between Caucasians and Asians: Asians have a greater tendency for obesity at a lower BMI than Caucasians [22]. This difference is linked to differences in adipokines such as adiponectin and leptin, which are associated with adiposity and muscle mass [23,24]. Therefore, ethnicity-specific equations are required for precise estimation of muscle mass by BIA. 

BIA devices have improved from single-frequency to multi-frequency and multi-segmental instruments using more electrodes, which has helped improve the accuracy [25]. Recently, more advanced technology has been used to evaluate both muscle mass and fat mass [26,27]. In this study, we investigated the accuracy of whole body muscle mass (WBMM) and appendicular skeletal muscle mass (ASMM) measures obtained with a BIA machine in subjects in the standing position for DXA reference values in Korean adults with a wide age range. In addition, we aimed to derive equations for better estimating muscle mass using relevant variables.

## 2. Methods

### 2.1. Subjects

We consecutively included 504 Koreans who had undergone both DXA and BIA over a short-term interval. Study participants were selected from the outpatient clinic of Seoul National University Bundang Hospital (SNUBH), Korea, from May 2015 to July 2017. We included physically active subjects aged 20–90 years. Subjects were excluded if they had severe debilitating diseases such as liver cirrhosis; stoke with paralysis; cerebral palsy; malignancy; a medical history of major operations, including organ removal and amputation; or were on hemodialysis or peritoneal dialysis. Written informed consent was received from all subjects. This study was approved by the Institutional Review Board of SNUBH (IRB no. B-1704-390-007) and complied with the principles of the Declaration of Helsinki and its contemporary amendments. 

### 2.2. Measurement of Clinical and Biochemical Parameters

Anthropometric parameters were measured in all study participants. Height (in cm) was measured to the nearest 0.1 cm and weight (in kg) was measured to the nearest 0.1 kg with the subject wearing light clothing. Body mass index (BMI) was calculated as weight (in kg) divided by height (in m) squared. Waist circumference was measured with the subject in a standing position at the umbilical point at the end of expiration by trained examiners. Blood pressure was measured using an automated blood pressure machine. Clinical data, including age, gender, comorbidity (including diabetes mellitus (DM), and hypertension), smoking habits, and alcohol consumption, were collected from each participant. Medication usage, such as diuretics and thiazolidinedione (TZD), was also investigated because these drugs can affect fluid status in the body. 

Blood samples were collected in the morning after 12 h fasting to measure biochemical variables such as fasting plasma glucose, blood urea nitrogen (BUN), creatinine, total protein, albumin, white blood cell, hemoglobin, hematocrit, platelet counts, lipid profiles, and liver enzymes. 

### 2.3. Muscle Mass Measurement Using DXA

For DXA, a Hologic Horizon W machine (Hologic Inc., Bedford, MA, USA) was used to measure WBMM and ASMM in the study participants. Before the DXA scan, participants were asked to remove all metal objects and to change into a gown. Scanning was performed with the subject supine, and the scan time was within 15 min. ASMM was calculated as the sum of the muscle mass in both arms and legs. 

### 2.4. Muscle Mass Estimation by BIA

For BIA, an Inbody770 machine (Inbody Co., Seoul, Korea) was used to estimate muscle mass [28,29]. The measurement was performed with the subject in a standing position grasping the electrodes with both hands abducted from the mid-body. There was a total of eight electrodes: two for each foot and two for each hand. The measurement comprised two combinations: *z*-axis at frequencies of 1, 5, 50, 250, and 500 kHz for impedance and *x*-axis at frequencies of 5, 50, and 250 kHz for reactance. Impedance was measured for five segments of the body: trunk, right and left arms, and right and left legs. Resistance was measured at four surface tactile electrodes placed on the dorsal surface of the hand and foot by the BIA generator; the formula V = ρχ height^2^/resistance was used to derive muscle mass mathematically [30]. Resistance is the resistance that occurs when alternating current passes through the body water, and reactance indicates the resistance of the cell membrane through which the alternating current passes. Impedance is the vector sum of these two components. In the InBody770 device, the reactance was calculated using a trigonometric formula with these parameters. Using the manufacturer’s algorithm, fat and muscle masses of the total body, arms, and legs were calculated separately. WBMM is the sum of the muscle masses in the whole body and ASMM is the sum of the lean mass of the arms and legs. The time gap between BIA measures and DXA scans was ~0.5 days. 

The InBody720, which had the same hardware as the InBody770, demonstrated a strong correlation with DXA (iDXA GE, Bedford, MA, USA) in ASMM (Pearson correlation coefficients 0.944 and 0.903, and standard error of estimate 1.051 kg and 0. 927 kg in men and women, respectively) [26]. A previous study showed a strong intra-class correlation (r = 0.9995) for the estimation of body fat percent using an InBody720 device [27]. Based on these data, InBody devices received a domestic medical device manufacturing item certificate by the Ministry of Food and Drug Safety of Korea.

Because BIA is sensitive to hydration status, participants were asked to refrain from alcohol consumption or vigorous exercise for 24 h before the measurement. Both BIA and DXA were measured in the morning after overnight fasting to make the hydration status as uniform as possible.

To minimize the contact noise, we cleaned the contacting surface of the electrodes with an alcohol swab before every measurement. In addition, the current electrode and voltage electrode were separated from each other by a total of eight electrodes because of the structure of the hand. Starting the measurement at the wrist and ankle, where the flow of current and measurement of voltage meet, minimized the influence of the finger and palm, which have high contact resistance. Using these methods, we were able to maintain high reproducibility in the body composition measurements using InBody devices [27].

### 2.5. Statistical Analysis

Data are presented as the mean ± standard deviation (SD) or number (*n*) with percentage. Pearson’s correlation analysis was used to test concordance between BIA and DXA muscle measurements. Agreement in muscle mass measurements between BIA and DXA was checked using the Bland–Altman method, but this was not used for cross-validation. To analyze differences among the subgroups according to age, gender, BMI, body fat, and other clinical features, we used the Student’s *t*-test and analysis of variance (ANOVA). We considered several possible models based on clinical importance and feasibility. Continuous variables were fitted with linear term or polynomial terms. Possible interaction terms were also included. A linear model was built with clinically relevant and statistically significant predictors, and linear or quadratic terms were considered during the model specification process. Normality assumptions were visually assessed through quantile–quantile plot, and multicollinearity of predictors was checked using a variance inflation factor. We derived the final model that had small Bayesian information criterion (BIC) value and not too many variables by parsimonious rule. For the final models, variance inflation factors (VIFs) were calculated to rule out collinearity. We used IBM SPSS Statistics v. 22.0 for Windows (IBM Corp., Armonk, NY, USA) and R statistical software v. 3.1.1 for Windows (Foundation for Statistical Computing, Vienna, Austria), and values of *p* < 0.05 were considered significant.

## 3. Results

### 3.1. Baseline Clinical Characteristics of the Study Population

The baseline characteristics of the subjects (*n* = 507) are shown in Table 1. The mean age of men (*n* = 213) and women (*n* = 294) was almost the same. For muscle mass assessment, men had a greater WBMM than women by about 13.0 kg either using the DXA (46.8 ± 6.5 kg vs. 34.0 ± 4.8 kg) or BIA methods (49.3 ± 6.6 kg vs. 36.1 ± 4.7 kg). The BIA approach estimated muscle mass to be greater than did DXA: 2.3 kg greater for WBMM and 2.0 kg greater for ASMM. Whole body fat mass and fat percentage assessed by BIA was greater in women than in men.

### 3.2. Comparison of Muscle Mass Estimated by BIA with That Measured by DXA (Table 2)

**Figure 1:**
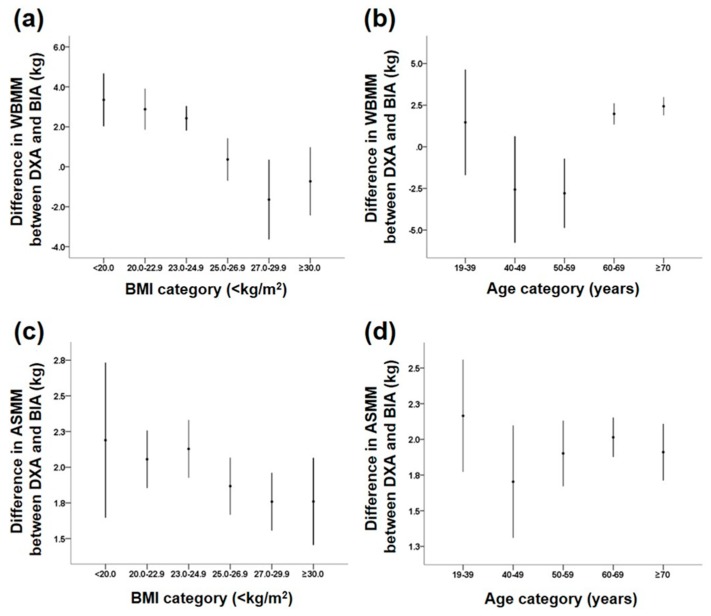
Differences in whole body lean muscle mass (WBMM) and appendicular skeletal muscle mass (ASMM) between DXA scans and BIA methods according to body mass index (BMI) and age categories. (**a**) WBMM by BMI group; (**b**) WBMM by age group; (**c**) ASMM by BMI group; and (**d**) ASMM by age group.

The WBMM and ASMM values estimated by BIA were highly correlated with those measured by DXA in the entire study group (both *r* > 0.97, *p* < 0.01). We then investigated whether differences between muscle masses measured by DXA and BIA were associated with anthropometric and biochemical parameters. Using ANOVA, the differences in WBMM between the two measurements were affected by gender, BMI, age, and body fat. Men had a larger difference in muscle mass assessments between the two methods than did women (*p* < 0.001). No gender differences were found in either the model using the interaction term gender or in the subgroup analysis stratified by gender (data not shown). Among the BMI categories, subjects with a lower BMI showed larger differences in WBMM assessments between the two methods, compared with those with a higher BMI (Figure 1). For age, the differences showed a U-shaped pattern: larger differences were found in people who were younger than 40 years and older than 60 years compared with those who were in their 40s and 50s.

For ASMM, the difference in its measurement between the two methods was found to be affected by gender: men showed larger differences than women (2.3 ± 1.1 kg vs. 1.8 ± 0.9 kg, *p* < 0.05) (Table 2). Like WBMM, subjects with a lower BMI showed larger differences in ASMM assessments between the two methods, compared with those with a higher BMI, but it had marginal significance (Table 2 and Figure 1). In contrast to WBMM, age group and body fat did not affect the differences in ASMM measurements between the two methods. 

**Figure 2:**
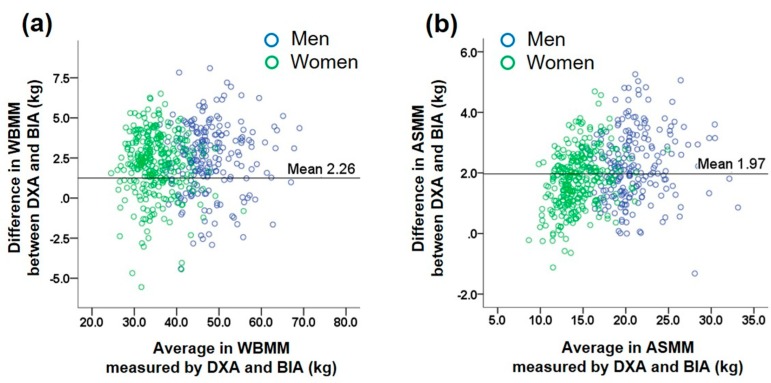
Bland–Altman plot for comparison between the two methods. (**a**) Whole body lean muscle mass (WBMM), (**b**) appendicular skeletal muscle mass (ASMM) in subjects who underwent DXA and BIA. Dotted horizontal lines are 95% confidence intervals.

The Bland–Altman plot for comparison between the two methods in the assessment of muscle mass showed a significant trend, which indicates increasing error with increase in muscle mass (Figure 2). We further investigated differences in WBMM and ASMM between the two methods according to subgroups classified as above or below a BMI of 25 kg/m^2^ and an age of 50 years in men and women to compare their differences according to these categories (Supplementary Table S1). In both men and women, individuals with BMI <25 kg/m^2^ had larger differences in both WBMM and ASMM estimates than did those with BMI ≥25 kg/m^2^ (all *p* < 0.01 except for *p* = 0.089 in women for WBMM). For the two age categories with a cutoff of 50 years, there were no significant trends in differences in WBMM and ASMM between the two methods. 

### 3.3. Subgroup Analysis of Mean Differences in Muscle Mass Estimates between DXA and BIA Methods According to Clinical Features (Table 3)

There were strong correlations for both WBMM and ASMM between the DXA and BIA methods (all *r* > 0.9, *p* < 0.001). For both WBMM and ASMM, hemoglobin levels, estimated glomerular filtration rate (eGFR), the presence of DM, and the use of diuretics or TZD did not affect the difference between the two methods.

### 3.4. Prediction of DXA Estimation of Muscle Mass Measured by BIA Method by Multivariate Regression Models (Table 4)

To best predict the DXA estimation of muscle mass by BIA measurement, we fitted multiple models and chose the model that satisfied minimal penalty functions while maintaining small variables and VIF <10. Finally, we generated equations using significant covariates including gender, BMI, and fat percent obtained by BIA. The final models were derived as follows:

WBMM = 4.01 + 0.28 × BMI + (−2.93) × Gender + 0.61 × WBMM-by-BIA + 0.001 × (WBMM-by-BIA)^2^ + 0.10 × fat percent-by-BIA;

ASMM = 5.07 + 0.26 × BMI + (−1.19) × Gender + 0.24 × ASMM-by-BIA + 0.01 × (ASMM-by-BIA)^2^ + (−0.06) × fat percent-by-BIA.

**Figure 3:**
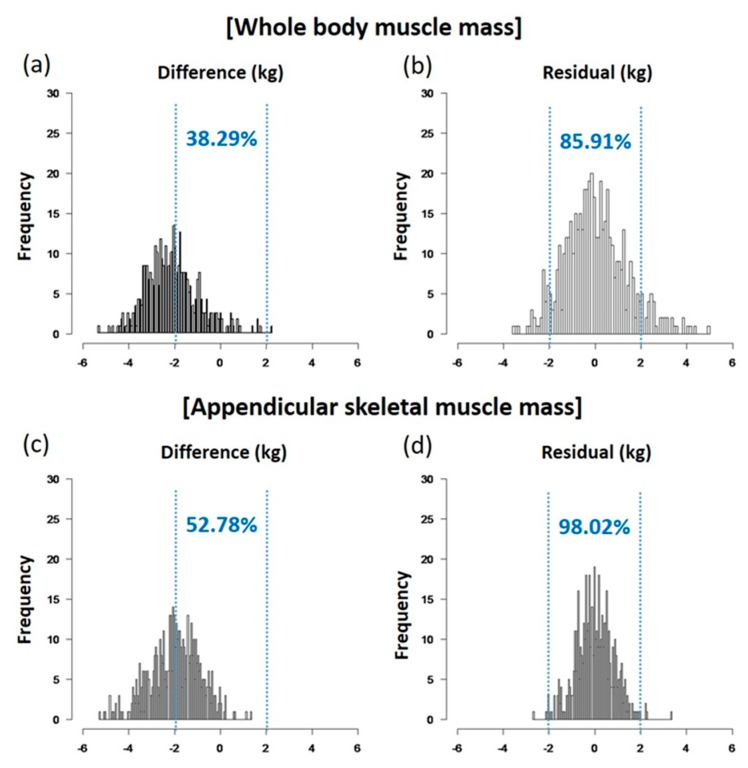
Comparing the percentages of residuals less than 2 kg between expectations before (**a**,**c**) and after (**b**,**d**) applying regression formulae. (**a**,**c**) WBMM; (**b**,**d**) ASMM.

Before and after applying the models, the differences between the two methods were compared based on the percentage of the value where the residual was less than 2 kg. After applying the model, for ASMM; the percentage of residuals <2 kg increased from 52.78% to 97.02% (Figure 3). As for WBMM, after applying the model, the percentage of residuals less than 2 kg increased from 38.29% to 85.91%. The effect sizes and 95% confidence intervals (CIs) for the difference in WBMM were 2.49 (2.22–2.76) for men and 2.12 (1.91–2.33) for women. The respective values for ASMM were 2.26 (2.10–2.41) for men and 1.75 (1.65–1.87) for women.

We also calculated VIF, the reciprocal of the tolerance statistics, and found that none of the VIF values were >10, which supports our interpretation of the reliable estimation of the coefficients. Therefore, including BMI and fat percent estimated by BIA improved the BIC without hampering the diagnostic of residuals. The leverage plots for ASMM and WBMM are shown in Supplementary Figure S1.

To validate the results from the regression models internally, we applied the bootstrapping method. The bootstrap standard errors of each variable were similar to those of the original models (Supplementary Table S2). The optimism average (bootstrap performance–test performance) values were also small (Supplementary Table S3). These data support the robustness of the original model. There was also no evidence of heteroscedasticity in the models when residual plots for ASMM and WBMM were checked visually (Supplementary Figures S2 and S3).

## 4. Discussion

In the present study, there was a high degree of correlation in muscle mass assessment between DXA and multifrequency-BIA methods in Korean adults both for WBMM and ASMM. The multifrequency-BIA method (Inbody770) overestimated the WBMM by 2.28 kg (95% CI 2.11–2.45) and ASMM by 1.97 kg (95% CI 1.87–2.06) compared with the DXA method (Hologic Horizon W).

Previous studies tried to validate BIA method in assessment of muscle mass with DXA. However, most studies were conducted with subjects of European origin [16,17]. In 1998, Pietrobelli et al. tried to validate a BIA estimate in the assessment of ASMM using height-adjusted appendicular impedance [31]. However, there were only 49 subjects, and inclusion of reactance failed to improve the estimation. In 2003, Kyle et al. tried to validate a prediction equation for estimating ASMM [16]. They suggested an equation based on reactance and resistance index (height^2^/R), and this improved the accuracy of ASMM estimation, particularly in older compared with younger subjects. However, they used a single frequency BIA machine. In 2015, Sergi et al. derived equations using resistance index and reactance for ASMM without considering age in their model [17]. Their equations showed an *r*^2^ value of 0.92 in the estimation of ASMM. They suggested that the resistive index was the single best predictor of ASMM in older people. However, in that study, subjects with chronic comorbidities were excluded, which could limit the use of this equation in sarcopenic elderly persons [17].

There have been several studies on Asian ethnic groups [32,33]. In a study on Japanese, a new BIA equation from DXA-measured ASMM explained 87% of the BIA values in men and 89% in women using an equation including an impedance index [32]. However, only 250 subjects were studied and only elderly people over 65 years old were recruited. Another study with 720 Koreans analyzed a new model including the Height^2^/resistance index for ASMM and found that it explained 82.5% of the variance [33]. However, they used an older BIA machine developed in 2002 and the study subjects were all elderly people aged over 65 years. 

More recently, there have been several studies investigating sarcopenia with BIA methods [26,34,35]. A study on 756 Japanese individuals aged 18–86 years attempted to generate the equations for ASMM using the traditional impedance index and an impedance ratio obtained from a BIA machine (MC-780A-N, TANITA, Tokyo, Japan) [34]. However, the correlation coefficients between ASMM values measured by DXA and those measured by BIA were unacceptable: 0.7 in men and 0.67 in women. Another study [35] showed that the accuracy of values from two advanced BIA machines differed: for the measurement of lean mass, there was 0.4 kg difference between DXA and an Inbody770 machine, but there was around a 5.5 kg difference between DXA and a TANIA980MA machine (TANITA). Another study on a Chinese population also found that the correlations between ASMM values obtained using the InBody720 (InBody, Seoul, Korea) BIA machine (which uses the same hardware as the InBody770) and those by DXA were high: the Pearson correlation coefficient and standard error of the estimate of the regression equation were 0.94 and 1.05 kg in men, and 0.90 and 0.93 kg in women, respectively [26].

In our study, with a wide age range, the actual differences between BIA and DXA estimates ranged from −5.55 to 8.10 kg for WBMM. We found that the difference in estimating WBMM between the two methods was affected by BMI, gender, and body fat. For example, subgroups with a lower BMI showed larger differences in muscle masses compared with their counterparts with a higher BMI. East Asians tend to have a lower muscle mass but higher fat percentage than Europeans for the same BMI [36]. The gap between BIA and DXA in Asian ethnic groups seems to be greater than that in Europeans or Africans [37]. These data suggest that ethnicity should be considered in the interpretation of BIA methods. From a different aspect, considering that WBMM includes trunk mass, which comprises much of the total muscle mass, more precise assessments in the estimations of muscle mass in the trunk or abdomen are needed. 

There were greater differences in muscle mass assessment between the two methods in men than in women. This result suggests than muscle mass assessment by BIA is affected by gender and supports the need for gender-specific formulae for analyzing body composition. Body fluid status or DM might also affect muscle mass estimations through alterations in impedance [38,39]. In our study, anemia, measures of renal function by eGFR, presence of DM, and the use of diuretics or TZD did not alter the difference between two methods, which allowed us to use the BIA approach in this population. 

### Strengths and Limitations

The strength of this study is that the number of study subjects was larger than in previous studies [15,16,17,31,32]. We included a much wider range of adults, aged from 28 to 89 years, and investigated ASMM as well as WBMM. To use the validation equation practically, we included variables that were relevant and easily accessible, such as age, gender, BMI, and body fat from the BIA measurements. With this simplified formula, we improved the accuracy of BIA in muscle mass assessment and minimized the percentage of residuals within 2 kg (Figure 3). This improvement in accuracy might be able to help researchers estimate muscle mass more accurately using BIA methods. In addition, we analyzed subgroups according to physiological and disease status, such as anemia, kidney function, DM, and medications such as diuretics or TZD, which were not considered in previous studies. We derived specific equations for ASMM as well as WBMM, which are critical in the diagnosis of sarcopenia [40,41]. 

However, there are several limitations to this study. First, this was not a community-based study and a non-probability convenience sampling method was used. Second, the participants were all Koreans. Therefore, the equations suggested in this study cannot be extrapolated to general populations or other ethnic groups and cross-validation is needed. However, we applied the bootstrapping method for the purpose of internal validation, and the result supports the robustness of the current study. Third, more variables are required to yield better accuracy of muscle mass estimation. To reduce the complexity of collecting additional information, we chose BMI, gender, and fat percentage data when building the regression equations because these can be easily obtained in clinical practice. Most companies selling BIA machines do not disclose the equations they use to estimate muscle mass, which makes it difficult to use them for research purposes [42]. We believe that incorporation of impedance values in regression equations is complicated and beyond the scope of this study. Fourth, because the BIA used in this study was developed for measuring body composition in the standing position, people who were bedridden, who had extremity amputation, or who could not hold the hand grip were not enrolled in this study. Lastly, the relative error was larger for ASMM measured by BIA than for WBMM measured by BIA, and this may have introduced a significant error in the assessment of sarcopenia based on appendicular skeletal muscle mass.

## 5. Conclusions

We found that muscle mass estimates by BIA were highly correlated with those measured by DXA. However, there was a tendency for the BIA method to overestimate muscle mass for DXA, particularly in men and in subjects with a low BMI. To increase accuracy in muscle mass assessment, we have developed regression equations with simple variables, which increased the percentage of residuals less than 2 kg to >85% in WBMM and >98% in ASMM. Although further validation is needed, we believe that these simplified and practical equations might offer an option to estimate muscle mass accurately at appendicular as well as in whole body levels.

## Figures and Tables

**Table 1:** Anthropometric and biochemical characteristics and comorbidity of the study populations (*n* = 507).

	Men (*n* = 213)	Women (*n* = 294)	* *p*
Age (years)	64.1 ± 1.3	63.4 ± 10.3	0.511
Height (cm)	168.6 ± 5.8	155.4 ± 5.6	<0.001
Weight (kg)	71.8 ± 11.0	60.9 ± 10.2	<0.001
BMI (kg/m^2^)	25.2 ± 3.1	25.2 ± 3.8	0.919
Waist circumference (cm)	88.9 ± 6.3	84.9 ± 8.9	<0.001
SBP (mmHg)	128.4 ± 13.7	127.2 ± 13.9	0.328
DBP (mmHg)	74.7 ± 10.1	75.2 ± 9.1	0.565
*Laboratory findings*			
FPG (70–110 mg/dL)	135.0 ± 41.5	117.6 ± 33.9	<0.001
HbA1c (4.0–6.4%)	7.1 ± 1.4	6.6 ± 1.2	<0.001
WBC (4–10 × 10^3^/μL)	6.4 ± 1.7	5.6 ± 1.6	<0.001
Hemoglobin (13–17 g/dL)	14.6 ± 1.5	13.2 ± 1.0	<0.001
Hematocrit (39–52%)	43.1 ± 4.1	39.7 ± 3.0	<0.001
Platelet (130–400 × 10^3^/μL)	211.5 ± 52.9	243.4 ± 53.9	<0.001
Total cholesterol (0–240 mg/dL)	161.1 ± 36.1	180.8 ± 40.2	<0.001
Triglycerides (0–200 mg/dL)	139.2 ± 90.5	132.4 ± 65.6	0.325
HDL-cholesterol (35–55 mg/dL)	47.5 ± 10.5	54.9 ± 11.9	<0.001
LDL-cholesterol (0–130 mg/dL)	91.6 ± 27.3	102.1 ± 29.9	<0.001
BUN (10–26 mg/dL)	17.4 ± 11.4	15.0 ± 4.3	0.001
Creatinine (0.70–1.40 mg/dL)	0.9 ± 0.2	0.7 ± 0.1	<0.001
eGFR (mL/min/1.73 m^2^)	85.3 ± 18.8	90.5 ± 19.3	0.003
Total protein (6.0–8.0 g/dL)	7.2 ± 0.4	7.3 ± 0.4	0.034
Albumin (3.3–5.2 g/dL)	4.4 ± 0.3	4.4 ± 0.2	0.053
AST (1–40 IU/L)	25.7 ± 8.3	27.2 ± 15.0	0.161
ALT (1–40 IU/L)	27.1 ± 13.8	26.1 ± 20.1	0.566
*Muscle mass by DXA*			
Whole body lean mass (kg)	46.8 ± 6.5	34.0 ± 4.8	<0.001
Appendicular skeletal muscle mass (kg)	19.9 ± 3.2	13.5 ± 2.2	<0.001
*Muscle mass by BIA*			
Whole body muscle mass (kg)	49.3 ± 6.6	36.1 ± 4.7	<0.001
Appendicular skeletal muscle mass (kg)	22.1 ± 3.3	15.3 ± 2.5	<0.001
*Fat mass by BIA*			
Fat mass (kg)	19.6 ± 5.7	22.5 ± 6.8	<0.001
Fat percent (%)	26.9 ± 5.7	36.4 ± 6.3	<0.001

Data are expressed as the mean ± SD. BMI, body mass index; WC, waist circumference; SBP, systolic blood pressure; DBP, diastolic blood pressure; WBC, white blood cell; FPG, fasting plasma glucose; LDL, low-density lipoprotein; HDL, high-density lipoprotein; BUN, blood urea nitrogen; eGFR, estimated glomerular filtration rate; AST, aspartate transaminase; ALT, alanine transaminase; DXA, dual-energy x-ray absorptiometry; BIA, bioelectrical impedance analysis. * *p* values by Student’s *t*-test between men and women.

**Table 2:** Mean differences in muscle mass observed by dual-energy X-ray absorptiometry (DXA) and bioelectrical impedance analysis (BIA) according to gender, BMI, and age groups and body fat categories.

	N	Mass by DXA (kg)	Mass by BIA (kg)	Difference (kg)	* *p*	^†^ *p*	ICC
(a) Whole body muscle mass (WBMM)							
Total	507	39.4 ± 8.4	41.6 ± 8.6	2.3 ± 2.0	<0.001		0.972
Gender						<0.001	
Men	213	46.8 ± 6.5	49.3 ± 6.6	2.5 ± 2.1	<0.001		0.947
Women	294	34.0 ± 4.8	36.1 ± 4.7	2.1 ± 1.9	<0.001		0.918
BMI (kg/m^2^)						<0.001	
<20	22	30.4 ± 4.7	33.8 ± 5.8	3.4 ± 2.1	<0.001		0.936
20–22.9	112	35.3 ± 6.7	38.5 ± 7.3	3.2 ± 1.7	<0.001		0.975
23–24.9	97	38.2 ± 6.9	41.3 ± 7.1	3.0 ± 1.6	<0.001		0.973
25–26.9	137	39.8 ± 7.0	41.7 ± 7.6	1.9 ± 2.0	<0.001		0.965
27–29.9	75	43.1 ± 9.0	44.6 ± 9.8	1.5 ± 1.8	<0.001		0.986
≥30	47	47.0 ± 10.2	47.5 ± 10.8	0.4 ± 2.1	<0.001		0.982
Age (years)						<0.001	
19–39	21	44.7 ± 12.5	47.7 ± 12.9	2.9 ± 1.9	<0.001		0.989
40–49	33	47.7 ± 9.5	49.3 ± 9.7	1.6 ± 1.7	<0.001		0.984
50–59	69	42.9 ± 8.7	44.5 ± 9.1	1.6 ± 2.5	<0.001		0.963
60–69	228	37.7 ± 7.5	40.3 ± 7.7	2.6 ± 1.9	<0.001		0.963
≥ 70	142	37.2 ± 6.8	39.5 ± 7.1	2.2 ± 2.0	<0.001		0.961
Body fat (%) ^‡^						0.003	
Non-obese	74	37.5 ± 8.0	40.5 ± 8.4	2.9 ± 2.0	<0.001		0.972
Obese	420	39.6 ± 8.5	41.7 ± 8.7	2.2 ± 2.0	<0.001		0.972
(b) Appendicular skeletal muscle mass (ASMM)							
Total	507	16.2 ± 4.1	18.2 ± 4.4	2.0 ± 1.1	<0.001		0.972
Gender						<0.001	
Men	213	19.9 ± 3.2	22.1 ± 3.3	2.3 ± 1.1	<0.001		0.939
Women	294	13.5 ± 2.2	15.3 ± 2.5	1.8 ± 0.9	<0.001		0.928
BMI (kg/m^2^)						0.093	
<20	22	12.2 ± 2.4	14.4 ± 3.1	2.2 ± 1.3	<0.001		0.932
20–22.9	112	14.4 ± 3.3	16.5 ± 3.9	2.1 ± 1.1	<0.001		0.973
23–24.9	97	15.8 ± 3.3	17.9 ± 3.7	2.1 ± 1.0	<0.001		0.968
25–26.9	137	16.3 ± 3.6	18.2 ± 3.9	1.9 ± 1.1	<0.001		0.958
27–29.9	75	17.8 ± 4.7	19.6 ± 5.0	1.8 ± 0.9	<0.001		0.985
≥30	47	19.5 ± 5.3	21.2 ± 5.4	1.8 ± 1.0	<0.001		0.981
Age (years)						0.503	
19–39	21	19.2 ± 6.5	21.3 ± 6.7	2.2 ± 0.9	<0.001		0.991
40–49	33	20.4 ± 4.7	22.1 ± 4.7	1.7 ± 1.1	<0.001		0.971
50–59	69	17.8 ± 4.2	19.7 ± 4.5	1.9 ± 1.0	<0.001		0.979
60–69	228	15.4 ± 3.6	17.5 ± 4.0	2.0 ± 1.0	<0.001		0.968
≥70	142	15.0 ± 3.3	17.0 ± 3.9	2.0 ± 1.2	<0.001		0.959
Body fat (%) ^‡^						0.675	
Non-obese	74	15.4 ± 3.8	17.4 ± 4.5	2.0 ± 1.1	<0.001		0.975
Obese	420	16.3 ± 4.2	18.2 ± 4.5	2.0 ± 1.1	<0.001		0.972

Data are expressed as the mean ± SD. * *p* values between DXA and BIA. ^†^ *p* values by one-way ANOVA for changes across groups. ^‡^ For men, non-obese (<25.7%), obese (≥25.7%); for women, non-obese (<35.9%), obese (≥36.0%).

**Table 3:** Pearson correlation coefficients between DXA and BIA muscle mass estimates in subgroups according to clinical features.

(a) Whole Body Muscle Mass (WBMM)						
		*n*	*BIA-DXA*	*r*	* *p*	^†^ *p*
Anemia	Hb ≥ 12 g/dL	457	2.33 ± 1.93	0.971	<0.001	0.734
	Hb < 12 g/dL	50	1.93 ± 2.26	0.938	<0.001	
Kidney function	eGFR ≥ 60 mL/min/1.73 m^2^	480	2.25 ± 1.91	0.975	<0.001	0.242
	eGFR < 60 mL/min/1.73 m^2^	27	2.80 ± 2.18	0.958	<0.001	
DM	DM (+)	327	2.16 ± 2.04	0.971	<0.001	0.157
	DM (−)	180	2.48 ± 1.70	0.978	<0.001	
Medication (1)	Diuretics (−)	457	2.32 ± 1.92	0.975	<0.001	0.187
	Diuretics (+)	50	1.83 ± 1.96	0.966	<0.001	
Medication (2)	TZD (−)	477	2.28 ± 1.92	0.973	<0.001	0.213
	TZD (+)	30	2.06 ± 1.72	0.989	<0.001	
**(b) Appendicular Skeletal Muscle Mass (ASMM)**						
Anemia	Hb ≥ 12 g/dL	457	2.01 ± 1.12	0.967	<0.001	0.617
	Hb < 12 g/dL	50	1.92 ± 1.12	0.956	<0.001	
Kidney function	eGFR ≥ 60 mL/min/1.73 m^2^	480	2.01 ± 1.11	0.973	<0.001	0.407
	eGFR < 60 mL/min/1.73 m^2^	27	2.32 ± 1.13	0.962	<0.001	
DM	DM (+)	327	2.16 ± 2.04	0.971	<0.001	0.697
	DM (−)	180	2.48 ± 1.70	0.973	<0.001	
Medication (1)	Diuretics (−)	457	2.03 ± 1.12	0.973	<0.001	0.704
	Diuretics (+)	50	1.92 ± 1.12	0.971	<0.001	
Medication (2)	TZD (−)	477	2.01 ± 1.13	0.971	<0.001	0.299
	TZD (+)	30	1.82 ± 1.14	0.973	<0.001	

* *p* values by Pearson correlation analysis between DXA and BIA. ^†^ *p* values by Fisher’s *z*-test between correlation coefficients. BIA, bioelectrical impedance analysis; DXA, dual-energy X-ray absorptiometry; Hb, hemoglobin; eGFR, estimated glomerular filtration rate; DM, diabetes mellitus; HbA1c, glycated hemoglobin; TZD, thiazolidinedione.

**Table 4:** Multivariate regression models for the prediction of DXA muscle mass measured by BIA.

Whole Body Muscle Mass				Appendicular Skeletal Muscle Mass			
	Co-Efficient	95% CI			Co-Efficient	95% CI	
		Lower	Upper			Lower	Upper
Intercept	4.01	0.80	7.22	Intercept	5.07	3.67	6.47
BIA-WBMM	0.61	0.48	0.74	BIA-ASMM	0.24	0.12	0.37
(BIA-WBMM)^2^	0.00	0.00	0.00	(BIA-ASMM)^2^	0.01	0.01	0.01
BMI	0.28	0.19	0.38	BMI	0.26	0.21	0.31
Gender	−2.93	−3.37	−2.48	Gender	−1.19	−1.45	−0.93
BIA-fat percent	0.10	0.05	0.15	BIA-fat percent	−0.06	−0.08	−0.04

WBMM, whole body muscle mass; ASMM, appendicular skeletal muscle mass; BIA, bioelectrical impedance analysis; DXA, dual-energy X-ray absorptiometry; CI, confidence interval.

## Supplementary Materials

The following are available online at http://www.mdpi.com/2072-6643/10/6/738/s1, Figure S1: The leverage plots for ASMM and WBMM, Figure S2: A residual plot for ASMM, Figure S3: A residual plot for WBMM, Table S1: Gender-specific subgroup analyses of mean differences between DXA and BIA muscle mass estimates according to BMI and age categories, Table S2: Bias-corrected percentile intervals of residuals in bootstrapping method, Table S3: Optimism average (bootstrap performance–test performance) values.
