# Supplementary material for: Comparison between Dual-Energy X-ray Absorptiometry and Bioelectrical Impedance Analyses for Accuracy in Measuring Whole Body Muscle Mass and Appendicular Skeletal Muscle Mass
Source: Nutrients. 2018 Jun 7;10(6):738. doi: 10.3390/nu10060738 (PMC6024648; doi:10.3390/nu10060738)
Supplement: Supplementary file 1 [file nutrients-10-00738-s001.pdf]

Supplementary Files

Table S1. Gender-specific subgroup analyses of mean differences between DXA and BIA muscle mass estimates according to BMI and age categories.

WBMM	*n*	DXA-WBMM (kg)	BIA-WBMM (kg)	BIA-WBMM – DXA-WBMM (kg)	*p	ICC	†p
**Men**							
BMI < 25 kg/m²	107	43.2 ± 3.9	46.5 ± 4.3	3.3 ± 1.8	<0.001	0.907	0.004
BMI ≥ 25 kg/m²	105	50.4 ± 6.7	52.1 ± 7.4	1.7 ± 2.2	<0.001	0.957	
Age < 50 years	23	56.6 ± 7.5	58.9 ± 7.8	2.3 ± 2.3	<0.001	0.957	0.215
Age ≥ 50 years	166	45.7 ± 5.4	48.3 ± 5.6	2.6 ± 2.2	<0.001	0.924	
**Women**							
BMI < 25 kg/m²	139	31.0 ± 3.0	33.9 ± 3.5	3.0 ± 1.6	<0.001	0.887	0.089
BMI ≥ 25 kg/m²	154	36.8 ± 4.6	38.1 ± 4.9	1.3 ± 1.9	<0.001	0.923	
Age < 50 years	31	39.1 ± 5.4	41.1 ± 5.3	2.0 ± 1.6	<0.001	0.954	0.336
Age ≥ 50 years	262	33.4 ± 4.4	35.5 ± 4.3	2.1 ± 2.0	<0.001	0.897	
**ASMM**	***n***	**DXA-ASMM (kg)**	**BIA-ASMM (kg)**	**BIA-ASMM – DXA-ASMM (kg)**	***p**	**ICC**	**†p**
**Men**							
BMI < 25 kg/m²	107	18.2 ± 1.9	20.8 ± 2.3	2.5 ± 1.1	<0.001	0.881	0.001
BMI ≥ 25 kg/m²	105	21.5 ± 3.4	23.5 ± 3.6	2.0 ± 1.1	<0.001	0.949	
Age < 50 years	23	25.0 ± 3.8	26.8 ± 3.9	1.9 ± 1.4	<0.001	0.937	0.520
Age ≥ 50 years	166	19.3 ± 2.6	21.7 ± 2.8	2.3 ± 1.1	<0.001	0.920	
**Women**							
BMI < 25 kg/m²	139	12.3 ± 1.4	14.1 ± 1.8	1.8 ± 0.9	<0.001	0.868	0.003
BMI ≥ 25 kg/m²	154	14.6 ± 2.3	16.3 ± 2.6	1.7 ± 1.0	<0.001	0.932	
Age < 50 years	31	16.1 ± 2.8	18.0 ± 2.8	1.9 ± 0.8	<0.001	0.960	0.433
Age ≥ 50 years	262	13.2 ± 1.9	15.0 ± 2.3	1.7 ± 1.0	<0.001	0.909	

Data are expressed as the mean ± SD. *p values by paired t test between DXA and BIA. †p values by Fisher's z-test between correlation coefficients. WBMM, whole body muscle mass; ASMM, appendicular skeletal muscle mass; BIA, bioelectrical impedance analysis; DXA, dual-energy X-ray absorptiometry.

Table S2. Bias-corrected percentile intervals of residuals in bootstrapping method.

For Whole Body Muscle Mass (WBMM)				
	Original	Boot Bias	Boot SE	Boot Med
(Intercept)	2.724	0.125	1.904	2.769
WBMM by BIA	0.649	-0.006	0.079	0.648
I × (WBMM by BIA^2)	0.002	0.000	0.001	0.002
BMI	0.249	0.001	0.052	0.249
Sex	-2.678	-0.014	0.230	-2.687
Fat percent by BIA	0.107	0.000	0.024	0.108
**For Appendicular Skeletal Muscle Mass (ASMM)**				
	Original	Boot Bias	Boot SE	Boot Med
(Intercept)	4.9049	-0.0246	0.6694	4.8770
ASMM by BIA	0.2664	0.0023	0.0607	0.2678
I × (ASMM by BIA^2)	0.0107	-0.0001	0.0014	0.0107
BMI	0.2568	0.0011	0.0246	0.2574
Sex	-1.1714	-0.0032	0.1312	-1.1747
Fat percent by BIA	-0.0592	-0.0003	0.0116	-0.0595

Table S3. Optimism average (bootstrap performance – test performance) values.

For Whole Body Muscle Mass (WBMM)						
	Original index	training	test	optimism	Corrected index	*n*
R-square	0.9735	0.9737	0.9732	0.0005	0.973	1000
MSE	1.872	1.8481	1.8931	-0.045	1.917	1000
G	9.3005	9.2833	9.2995	-0.0163	9.3167	1000
Intercept	0	0	0.0057	-0.0057	0.0057	1000
Slope	1	1	0.9999	0.0001	0.9999	1000
**For Appendicular Skeletal Muscle Mass (ASMM)**						
	Original index	training	test	optimism	Corrected index	*n*
R-square	0.9569	0.9571	0.9564	0.0007	0.9562	1000
MSE	0.7309	0.7226	0.7392	-0.0166	0.7475	1000
g	4.5452	4.5315	4.5447	-0.0132	4.5584	1000
Intercept	0	0	-0.0037	0.0037	-0.0037	1000
Slope	1	1	1.0003	-0.0003	1.0003	1000

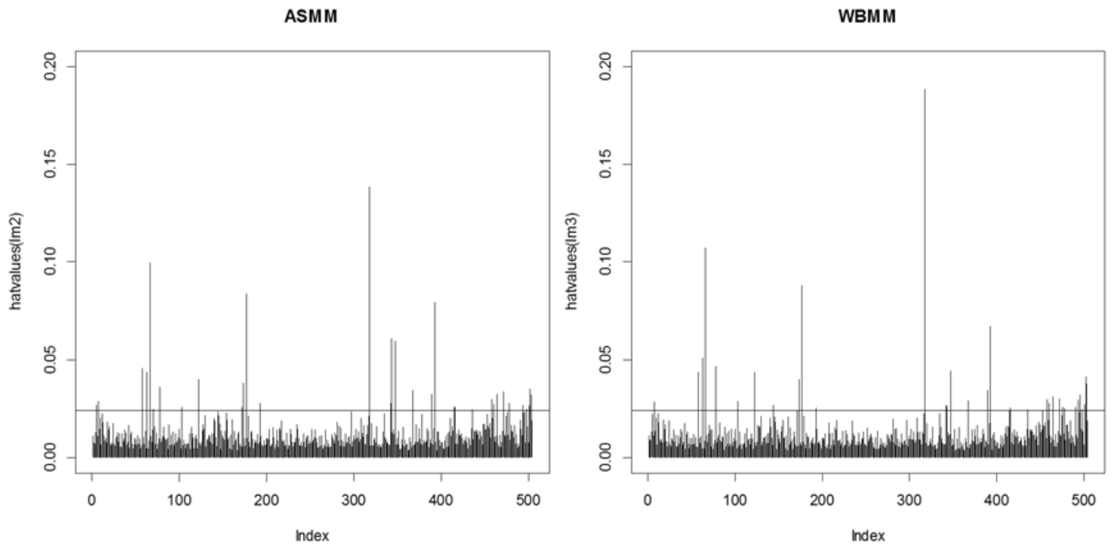

Figure S1. The leverage plots for ASMM and WBMM.

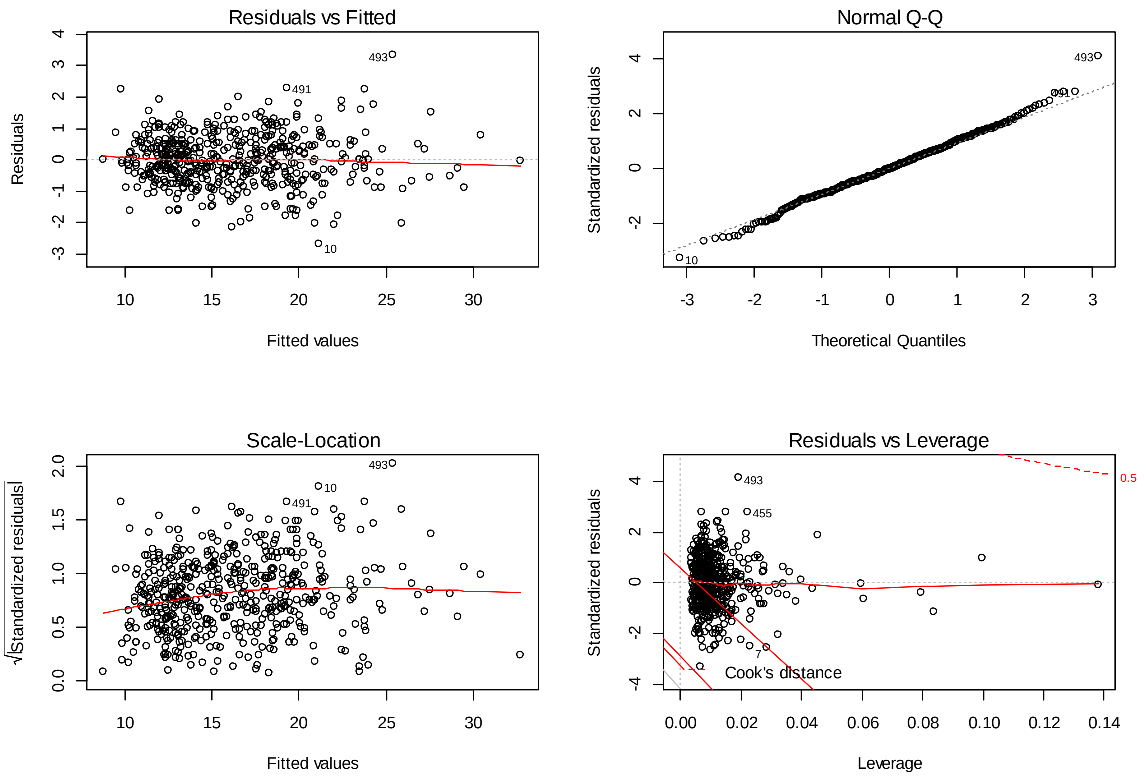

Figure S2. A residual plot for ASMM.

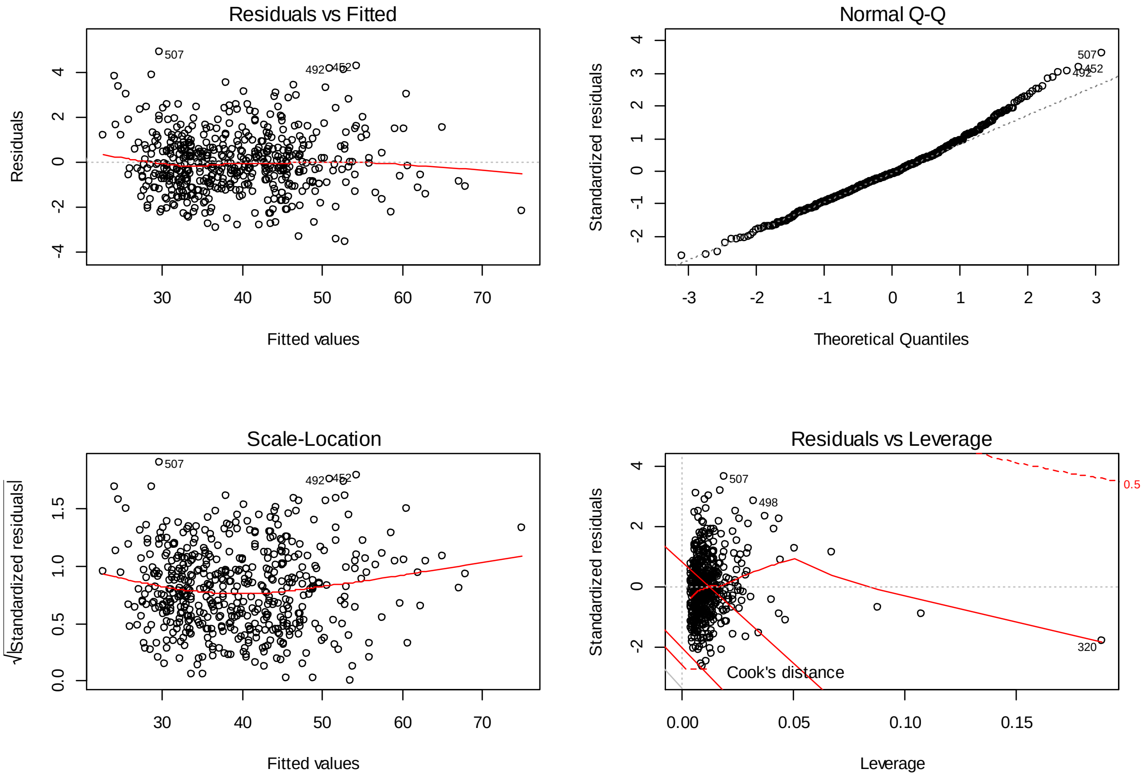

Figure S3. A residual plot for WBMM.
